# Exercise-induced Activation of SIRT1/BDNF/mTORC1 Signaling Pathway: A Novel Mechanism to Reduce Neuroinflammation and Improve Post-stroke Depression

**DOI:** 10.62641/aep.v53i2.1838

**Published:** 2025-03-05

**Authors:** Junze Tang, Lina Lu, Jiabo Yuan, Lin Feng

**Affiliations:** ^1^Graduate School, Harbin Sport University, 150008 Harbin, Heilongjiang, China; ^2^Department of Rehabilitation Medicine, The Second Affiliated Hospital of Heilongjiang University of Chinese Medicine, 150000 Harbin, Heilongjiang, China; ^3^Graduate School, Heilongjiang University of Chinese Medicine, 150000 Harbin, Heilongjiang, China

**Keywords:** post-stroke depression, neuroinflammation, exercise, SIRT1/BDNF/mTORC1

## Abstract

**Background:**

Neuroinflammation and neurogenic disorders lead to depression in stroke patients. As, exercise intervention, a non-drug therapy, has been proven effective in post-stroke depression (PSD) patients. However, the underlying molecular mechanism by which exercise improves PSD still needs to be explored. Therefore, utilizing the mice model, this study aimed to observe the pathological changes in PSD and to investigate the mechanism by which exercise improves PSD symptoms.

**Methods:**

A middle cerebral artery occlusion (MCAO)+chronic unpredictable mild stress (CUMS) method was used to establish the PSD mice model, and the model mice were subjected to exercise interventions. Behavior tests were conducted to validate changes in depression-like behaviors. Western blot and reverse transcription-polymerase chain reaction (RT-qPCR) analyses were used to evaluate the expression levels of silent information regulator factor 2-related enzyme 1 (SIRT1), brain-derived neurotrophic factor (BDNF), and mammalian target of rapamycin complex 1 (mTORC1) signaling pathway in brain tissue. Enzyme linked immunosorbent assay (ELISA) analyses were performed to assess the effects of exercise on neuroinflammatory markers. Hematoxylin-Eosin (HE) and Nissl staining were used to examine exercise-induced histopathological change in the brain tissue. Furthermore, SIRT1 was knocked down using an adenovirus-mediated approach, and glial fibrillary acidic protein (GFAP) staining was used to determine the number of astrocytes in brain tissue.

**Results:**

Exercise significantly alleviates the symptoms of neurological dysfunction in model mice (*p* < 0.01). Exercise decreased the immobile time of PSD mice (*p* < 0.05) and increased the total exploration distance and crossing area (*p* < 0.05). Furthermore, exercise significantly reduced inflammatory marker levels, such as interleukin (IL)-6, tumor necrosis factor-alpha (TNF-α), and IL-1β (*p* < 0.05), and elevated anti-inflammatory factor IL-10 levels (*p* < 0.01). Moreover, exercise training alleviated inflammatory infiltration, increased the number of Nissl bodies (*p* < 0.05), and improved pathological changes in PSD mice. Additionally, exercise enhanced the expression levels of SIRT1, BDNF (*p* < 0.01), synaptophysin (Syn1), and postsynaptic density (PSD) 95 (*p* < 0.01), thereby improving synaptic plasticity and enhancing astrocyte activity (*p* < 0.05). Furthermore, compared to the model+exercise+con-shRNA group, SIRT1 knockdown inhibited protein expression in the mammalian target of rapamycin (mTOR) pathway (*p* < 0.05), reversing exercise-induced effects.

**Conclusion:**

Exercise intervention reduces post-stroke depression-like behavior by activating SIRT1/BDNF/mTORC1 signaling pathway and reducing neuroinflammation. These findings provide insights into understanding the role of exercise in treating post-stroke depression and offer a theoretical basis for developing novel antidepressant strategies.

## Introduction

Post-stroke depression (PSD) is a complex mental health condition characterized by reduced activity, anhedonia, and neurovegetative symptoms, which significantly affect cognitive and social functioning and can lead to severe suicidal tendencies [1]. PSD has become a leading cause of disability globally. Key biological disturbances resulting from excessive inflammatory stimulation of the brain include disruptions to neuroplasticity pathways, and alterations in the function and morphology of glial cells [2]. Current treatment approaches primarily include antidepressants, adjuvant medications, non-pharmacological treatments, evidence-based psychotherapies, and physical non-drug therapies. However, the prolonged use of antidepressants is significantly linked to drug resistance, contributing to increased suicide rates [3, 4], and many patients undergoing psychological treatments fail to achieve sufficient relief. Therefore, there is an urgent need for innovative and effective treatment strategies.

Exercise therapy has demonstrated promise in reducing the incidence of psychiatric disorders, such as PSD. The advantages of exercise therapy include simplicity, no side effects, low cost, and optimum for patients resistant to medication [5]. Exercise alleviates PSD-like behaviors through various mechanisms, including modulating neurotrophic factors, decreasing neuroinflammation, and enhancing synaptic plasticity [6]. A key focus has been the activation of the silent information regulator factor 2-related enzyme 1 (SIRT1) signaling pathway, which emerges as a novel therapeutic target for treating depression.

SIRT1, a highly conserved nicotinamide adenine dinucleotide (NAD)-dependent deacetylase, deacetylates numerous substrates, including transcription factors, histones, and various enzymes. Furthermore, SIRT1 regulates various physiological processes, such as apoptosis, cell differentiation, development, autophagy, and cancer [7]. When activated, SIRT1 shows anti-inflammatory features, counteracts depression-related phenotypes, and inhibits chronic stress-induced abnormal dendritic impairments; however, excessive inflammation and organ damage impede SIRT1 activity [8].

Furthermore, brain-derived neurotrophic factor (BDNF) is crucial for neuronal survival, growth, and synaptic plasticity. Its levels are typically reduced during depression, while in the hippocampus, it modulates synaptic efficacy by altering presynaptic transmitter release or increasing postsynaptic transmitter sensitivity, thus inducing long-term increases in synaptic plasticity [9, 10]. Moreover, the mammalian target of rapamycin complex 1 (mTORC1), a regulator of cell proliferation and metabolism, plays a crucial role in synaptic structural and functional plasticity [11]. Depression patients usually show alleviated mTORC1 expression levels in the prefrontal cortex, disrupting synaptic formation. When stimulated by factors such as tumor necrosis factor-alpha (TNF-α), receptor activator of nuclear factor kappa-B ligand (RANKL), and BDNF, mTORC1 serves as a downstream regulator of exercise [12]. The complex interactions among these three components form a network influencing neurogenesis, synaptic function, and inflammatory responses.

This study aimed to explore the mechanism of action of exercise, a non-pharmacological therapy, in reducing neuroinflammation and depression-like behaviors in PSD. It specifically assessed the effects of exercise on neuroglial cell activity and the advantages of activating the SIRT1/BDNF/mTORC1 signaling pathway on cognition and neural growth in mice, offering insights into potential targets for treating PSD.

## Materials and Methods

### Selection and Acclimatization of Mice

Male C57BL/6 mice (n = 40), 8-week-old and weighing 18 to 22 g, were purchased from the Laboratory Animal Centre of Shanghai (SLAC Laboratory Animal Co. Ltd., Shanghai, China). They were acclimatized and fed for 1 week at a steady temperature (22 °C) and humidity (70%), with a 12-hour light-dark cycle. The study design was approved by the Medicine Ethics Organization of the Second Affiliated Hospital of Heilongjiang University of Chinese Medicine (approval number: 2024029).

### Development of Stroke Mice Model

Mice were randomly divided into the control group (Con), the PSD model group (M), and the PSD model+exercise group (M+Exe), with 8 mice per group. Furthermore, two separate PSD model+exercise mice groups were designated for adeno-associated virus injection. To simulate stroke, the middle cerebral artery occlusion (MCAO) model was established using the suture-occlusion method [13]. Under general anesthesia with 1% pentobarbital sodium at a dose of 50 mg/kg, the left common carotid artery (CCA) and external carotid artery (ECA) were exposed. A coated nylon filament was inserted through the ECA incision and advanced to block the middle cerebral artery (MCA). After 60 minutes, the thread was removed to restore the blood flow. The control group underwent arterial exposure without filament insertion. After the procedure, the mice were housed and cared for in a warm environment, and the chronic unpredictable mild stress (CUMS) procedure was performed 3 days later.

### Assessment of Neurological Deficits

The neurological function of the mice was assessed using the Longa score [14, 15]: where 0 points show no neurological deficits, 1 point indicates inability to fully extend the front paw on the paralyzed side, 2 points show turning toward the paralyzed side while walking, 3 points means tilting to the paralyzed side during walking, and 4 points indicates loss of automatic walking ability accompanied by loss of consciousness. Higher scores indicate severe neurological deficits. Mice with neurological defect score >1 were selected for CUMS procedure.

### CUMS Procedure

The CUMS model is widely utilized to mimic the long-term effects of chronic stress and induce depressive-like states in mice. After the adaptation period, mice were treated with 10 different stressors over a 4-week, with two or three stressors randomly applied daily, ensuring no repetition within a 3-day interval [16]. The stressors included tail clamping (10 minutes), reversed light-dark cycles (24 hours), cage tilting at a 45° angle (12 hours), physical restraint (6 hours), wet bedding (24 hours), food and water deprivation (24 hours), 45 °C heat stimulation (5 minutes), continuous light exposure (12 hours), noise stress (3 hours), and swimming in 4 °C water (5 minutes).

### Exercise Procedure

The mice underwent a 7-day adaptive treadmill training. The treadmill was inclined at 0°, and the mice ran at a speed of 10 m/min for 10 min daily. After the adaptive treadmill training phase, the training continued for 6 days, with the duration increased by 10 min per day. The mice in the exercise group were engaged in regular exercise, running for 60 min at a speed of 10 m/min per day, 6 days per week, for 4 weeks [17].

### Behavioral Tests

Mice underwent different behavioral assessments such as Forced Swimming Test (FST) [18], Open Field Test (OFT) [19], and Tail Suspension Test (TST) [20].

During FST, each mouse was placed in a clear glass cylinder (15 cm diameter, 30 cm height) filled with water to a depth of 15 cm at 23 ± 1 °C. Immobility during the last 4 minutes of the 6-minute session was recorded, with immobility defined as floating motionlessly while keeping the head above water for breathing.

In OFT, mice were placed in the center of a 50 × 50 × 40 cm box, and their activities were recorded for 5 minutes using a camera (Ethovision XT, Noldus, Wageningen, Netherlands). Measurement included the total distance traveled and the number of cells crossed. The box was thoroughly cleaned after each trial.

**Fig. 1.**
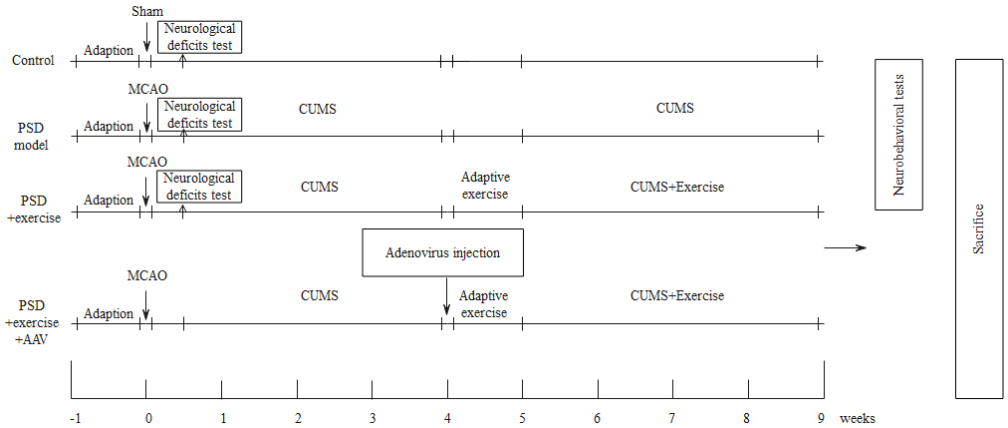
**Grouping and experiment design for behavioral assessment of the mice**. Note: PSD, post-stroke depression; MCAO, middle cerebral artery occlusion; AAV, adeno-associated virus; CUMS, chronic unpredictable mild stress.

During TST, mice were suspended 40 cm above the ground by adhesive tape placed 1.5 cm from the tail tip for 6 minutes. Immobility time during the last 4 minutes was recorded. The grouping and experimental procedures are shown in Fig. 1.

### Histomorphological Examination

Mice were rapidly decapitated, and brain tissues, especially from the hippocampal region, were collected for subsequent histomorphological analyses.

For Nissl staining, mice brain tissues were dewaxed, hydrated, and then embedded in paraffin. The embedded tissues were sliced into 6-µm sections, stained with cresyl violet (BL1039A, Biosharp, Hefei, China) for 5 min at 56 °C, dehydrated using gradient alcohol solution, transparent in xylene for 5 min, and sealed with neutral balsam.

During the Hematoxylin-Eosin (HE) staining procedure, the prepared sections were stained with hematoxylin (BL700A, Biosharp, Hefei, China) for 3 minutes, fractionated in 1% hydrochloric acid for 10 seconds, and then stained in eosin stain for 3 minutes. After that, the sections were dehydrated with gradient alcohol, cleared in xylene for 5 minutes, and sealed with neutral adhesive.

Finally, images were captured using a light microscope (Eclipse Ci-L, Nikon, Tokyo, Japan), and image processing was conducted employing ImageJ version 1.53k software (NIH, Bethesda, MD, USA).

### Enzyme Linked Immunosorbent Assay (ELISA)

The levels of inflammatory factors, including interleukin (IL)-6 (MM-0163M2), TNF-α (MM-0132M2), IL-1β (MM-0040M2), IL-10 (MM-0176M2), and BDNF (MM-0204M2), in brain tissue were measured using ELISA kits (Meimian Industrial, Nanjing, China). During these assays, standard solution and samples were added to the wells of a pre-coated microtiter plate and incubated for 30 minutes. After washing 5 times with washing buffer, horseradish peroxidase (HRP)-conjugated antibody was added to each well, followed by incubation for 30 minutes. Plates were washed again, and substrate solution was applied, allowing the color to develop in 10 minutes. Finally, the reaction was terminated by adding a termination solution.

The absorbance was determined at a wavelength of 450 nm using a microplate reader (1681130, Bio-Rad, Hercules, CA, USA). The concentration of the target antigen in the sample was calculated based on the standard curve.

### Adenovirus Vector Construction and Injection

SIRT1 knockdown was constructed using the GV478 U6-MCS-CAG-EGFP adeno-associated virus (AAV). The shRNA sequence targeting SIRT1 was 5^′^-GATGAAGTTGACCTCCTCA-3^′^, while the control shRNA sequence was 5^′^-TTCTCCGAACGTGTCACGT-3^′^. The shRNA sequences were cloned into vectors, and the virus was concentrated, purified, and its titer was determined using quantitative polymerase chain reaction (PCR). Furthermore, the viral titer was diluted to 5 × 10^12^ before starting the experiment. These procedures were performed by Genechem Co. Ltd., Shanghai, China. For stereotaxic microinjection, mice were anesthetized with 1% sodium pentobarbital at a dose of 50 mg/kg and fixed in a stereotaxic frame. After exposing the skull, a catheter was placed into the hippocampus and fixed with dental cement. Following this, 1 µL of AAV-SIRT1 shRNA or AAV-control shRNA was infused at 0.2 µL/min using a Hamilton syringe (87900, Hamilton, NV, USA). The needle was held in place for 5 min to allow the virus to spread. The incision was then aseptically sutured.

### Reverse Transcription-Polymerase Chain Reaction (RT-qPCR)

Total RNA was extracted from the hippocampus using TRIzol reagent (AM9738, Thermo Fisher Scientific, Inc., Waltham, MA, USA) and reverse transcribed to cDNA employing the PrimeScript™ RT premix kit (RR092S, Takara Bio, Tokyo, Japan). Real-time quantitative PCR (CN830S, Takara Bio, Tokyo, Japan) was performed using SYBR Green premix to quantify the transcription levels of target genes. The relative gene expression was assessed using the 2^-Δ⁢Δ⁢Ct^ method, with glyceraldehyde-3-phosphate dehydrogenase (GAPDH) as the internal control. The primer sequences used in qPCR are as follows: BDNF: Forward 5^′^-CAGGACAGCAAAGCCACAAT-3^′^, Reverse 5^′^-GCCTTCATGCAACCGAAGTA-3^′^; SIRT1 1: Forward 5^′^-AAAGGAATTGGTTCATTTATCAGAG-3^′^, Reverse 5^′^-TTGTGGTTTTTCTTCCACACA-3^′^; Postsynaptic density 95 (PSD95): Forward 5^′^-ATGTGCTTCATGTAATTGACGC-3^′^, Reverse 5^′^-TTTAACCTTGACCACTCTCGTC-3^′^; Synaptophysin (Syn1): Forward 5^′^-ACAGCAGTGTTCGCTTTCA-3^′^, Reverse 5^′^-CAGAGCACCAGGTTCAGG-3^′^; GAPDH: Forward 5^′^-AGGTCGGTGTGAACGGATTTG-3^′^, Reverse 5^′^-TGTAGACCATGTAGTTGAGGTCA-3^′^.

### Western Blot (WB)

Hippocampal tissues were homogenized using a tissue grinder and lysed on ice in a protein lysate containing protease inhibitors and phosphatase inhibitors (BC3710, Solarbio, Beijing, China) for 10 minutes. Protein concentration was determined using a bicinchoninic acid assay (BCA) protein assay kit (P0012, Beyotime, Shanghai, China). Proteins from each sample were separated by sodium dodecyl sulfate-polyacrylamide gel electrophoresis (SDS-PAGE) and transferred onto a polyvinylidene difluoride (PVDF) membrane (IPVH00010, Merck Millipore, Billerica, MA, USA) pre-activated with anhydrous methanol. After this, the membranes were blocked with 5% skim milk for 1 h at room temperature and underwent overnight incubation at 4 °C with the following primary antibodies: SIRT1 (1:1000, 13161-1-AP), BDNF (1:1000, 25699-1-AP), Syn1 (1:5000, 17785-1-AP), PSD95 (1:5000, 30255-1-AP), protein kinase B (Akt) (1:2000, 10176-2-AP), phosphorylated-protein kinase B (p-Akt) (1:2000, 66444-1-Ig), mammalian target of rapamycin (mTOR) (1:5000, 66888-1-Ig), phosphorylated-mammalian target of rapamycin (p-mTOR) (1:5000, 67778-1-Ig), tropomyosin receptor kinase B (TrkB) (1:1000, 13129-1-AP), GAPDH (1:5000, 10494-1-AP) (Proteintech, Wuhan, China), phosphorylated-tropomyosin receptor kinase B (p-TrkB) (1:500, ABN1381, Merck), phosphoinositide 3-kinase (PI3K) (1:1000, ab40776, Abcam, Shanghai, China), and phosphorylated-phosphoinositide 3-kinase (p-PI3K) (1:500, ab182651, Abcam). The following day, the membranes were washed 5 times with 1× tris buffered saline with tween 20 (TBST) and subsequently incubated with HRP-conjugated secondary antibodies goat anti-rabbit immunoglobulin G (IgG) (1:5000, SA00001-2, Proteintech, Wuhan, China) or goat anti-mouse IgG (1:5000, SA00001-1, Proteintech, Wuhan, China) at room temperature for 2 hours. The protein gray value was determined using an enhanced chemiluminescence (ECL) detection system (P0018, Beyotime, Shanghai, China).

### Glial Fibrillary Acidic Protein Staining

The hippocampus was fixed in 10% formaldehyde overnight. The tissue samples were gradually dehydrated with ethanol solutions of different concentrations, followed by clearing and soaking in wax. They were then embedded in paraffin wax and sectioned into 4-µm slices. In the next step, antigen retrieval was conducted by boiling. The tissue sections were blocked with bovine serum albumin (BSA) at room temperature for 1 hour, followed by overnight incubation at 4 °C with the primary glial fibrillary acidic protein (GFAP) antibody (Z0334; Agilent Dako, Santa Clara, CA, USA), dilution 1:400. The following day, the tissue sections were washed 3 times with phosphate buffered saline (PBS) and underwent incubation with the secondary antibody (1:5000, ab205718, Abcam, Shanghai, China) at room temperature for 2 hours. After this, the sections were visualized using 3, 3^′^-diaminobenzidine tetrahydrochloride (DAB), followed by counterstaining with hematoxylin. The sections were sealed, and the staining results were observed and photographed employing the OlyVIA VS200 imaging system (Olympus, Tokyo, Japan).

### Statistical Analysis

Statistical analyses were performed using Graph-Pad Prism 8.0 Software (Graph-Pad Software, San Diego, CA, USA). Data were expressed as mean ± standard deviation. The Shapiro-Wilk test was applied to evaluate the normality of the data distribution. Comparison among multiple groups was conducted using a one-way analysis of variance (ANOVA), followed by Fisher’s least significant difference test for post hoc comparison. A *p*-value of <0.05 indicated a statistically significant difference.

## Results

### Establishment of Stroke Model in Mice

The stroke mice were successfully established. The neurological deficit scores of the mice in the model group were all greater than 1, and obvious neurological dysfunction was found after MCAO intervention (*p* < 0.01) (Table 1).

**Table 1.** **Neurological deficit scores among different mice groups (mean ± SD)**.

Group	Con	M	M+Exe
n	8	8	8
Before intervention	0	0	0
After intervention	0	2.25 ± 0.70^*⁣**^	1.45 ± 0.21^#⁢#^

Note: Con: the Control group, M: the CUMS model group, M+Exe: the CUMS model+exercise group. ^*⁣**^p < 0.001 versus the control group; ^#⁢#^*p* < 0.01 versus the model group. SD, standard deviation.

### Exercise Alleviates Depression-like Behavior in Post-stroke Depressed Mice

**Fig. 2.**
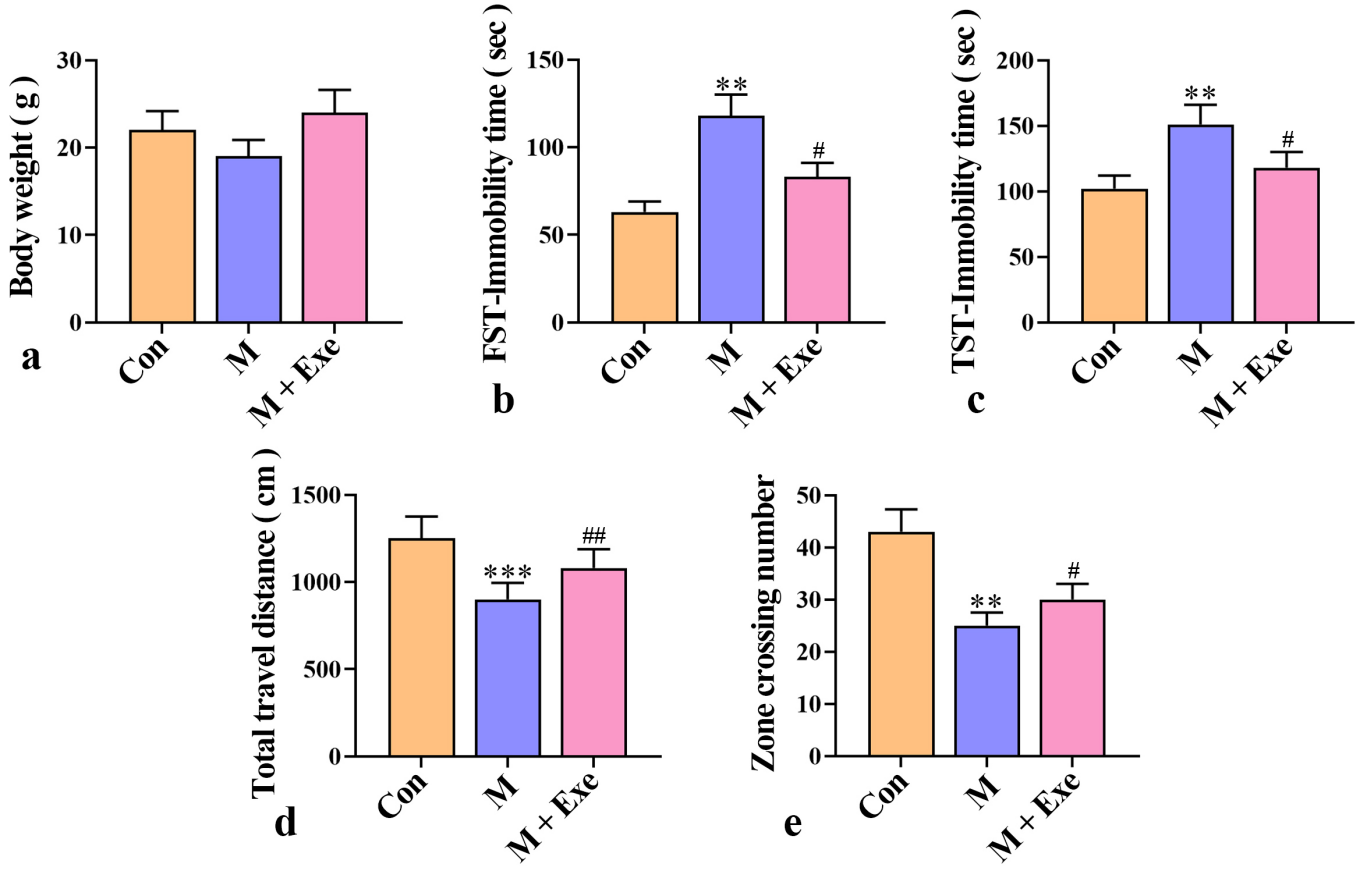
**Neurobehavioral detection of depression-like behavior in mice (n = 8)**. (a) Body weight of mice in each group. (b) Immobility time in FST. (c) Immobility time in TST. (d) Total distance traveled in OFT. (e) Number of crossed grids in OFT. Note: FST, Forced Swimming Test; TST, Tail Suspension Test; OFT, Open Field Test. ^*⁣**^p < 0.001, ^**^p < 0.01 versus the control group; ^#⁢#^*p* < 0.01, ^#^*p* < 0.05 versus the model group.

Following exercise training, no significant change in body weight was observed in model mice (Fig. 2a). Furthermore, behavioral tests were used to assess changes in depression-like behaviors. Immobility time in the FST and TST indicated the levels of desperation in mice. As shown in Fig. 2b,c, the immobility time was significantly prolonged in post-stroke depressed mice (*p* < 0.01). However, exercise training significantly reduced immobility time (*p* < 0.05). These results suggest that exercise alleviated despair and depression-like behaviors in the mice.

The total distance of movement in the OFT was used to evaluate the dynamic state of the mice, and the number of areas crossed reflected their exploratory behavior. As depicted in Fig. 2d,e, compared to the control group, the total movement distance and the number of crossed areas were significantly reduced in the model group (*p* < 0.01). However, these measures were significantly increased after exercise training (*p* < 0.05), indicating that exercise enhanced vitality and exploratory behavior and alleviated depressive symptoms.

### Exercise Alleviates Neuroinflammation in Post-stroke Depressed Mice

**Fig. 3.**
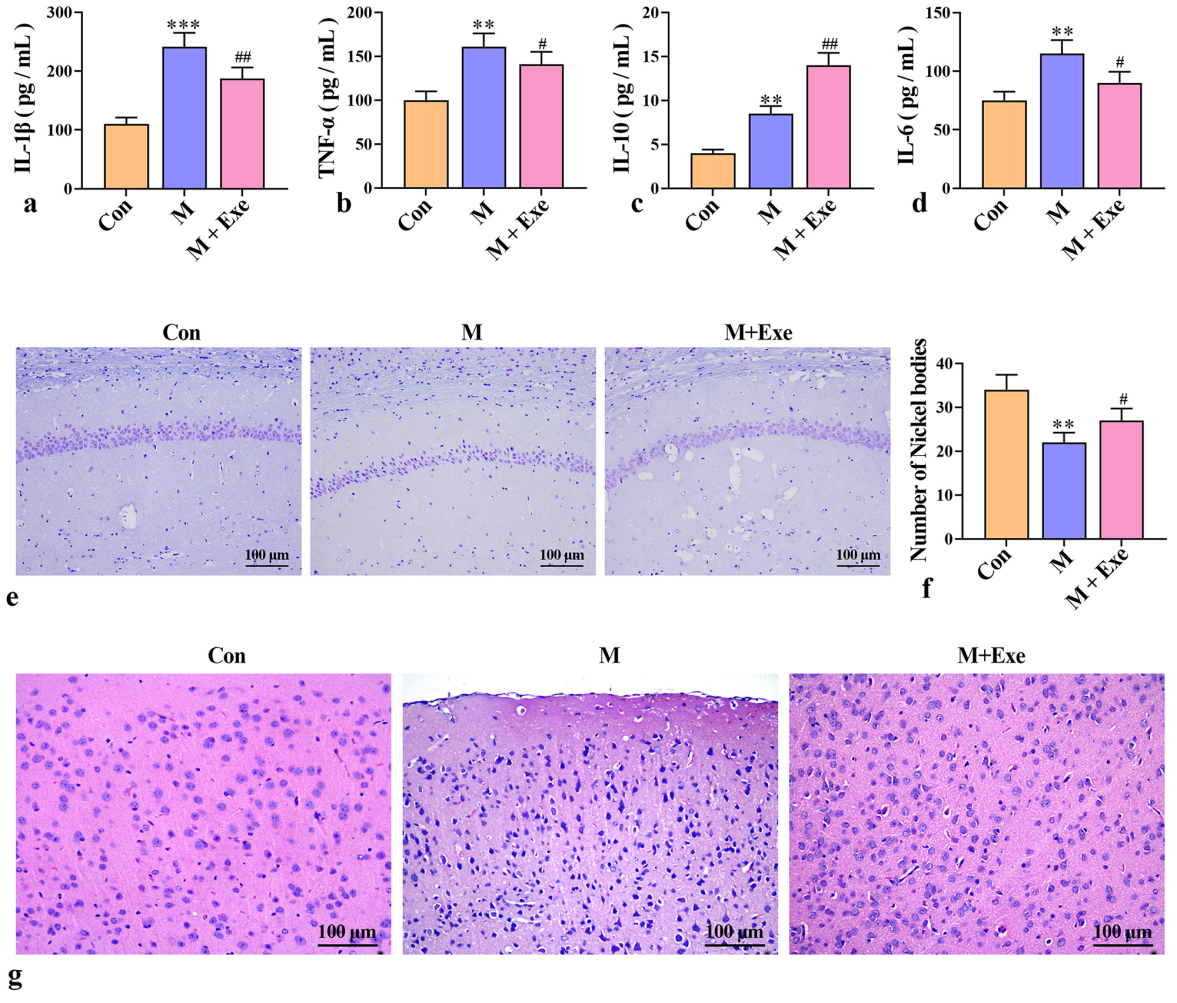
**Exercise reduces neuroinflammation and nerve damage (n = 8)**. (a) IL-1β levels in mouse brain tissue. (b) TNF-α levels in mouse brain tissue. (c) IL-10 levels in mouse brain tissue. (d) IL-6 levels in mouse brain tissue. (e,f) Representative images of neuron morphology detected by Nissl staining. (g) Representative images of neuroinflammation detected by HE staining. ^*⁣**^p < 0.001, ^**^p < 0.01 versus the control group; ^#⁢#^*p* < 0.01, ^#^*p* < 0.05 versus the model group. IL, interleukin; TNF-α, tumor necrosis factor-alpha; HE, Hematoxylin-Eosin.

To evaluate the degree of neuroinflammation, the levels of pro-inflammatory cytokines (IL-6, TNF-α, and IL-1β) and anti-inflammatory cytokine (IL-10) in mouse brain tissue were determined using ELISA. As shown in Fig. 3a–d, both pro-inflammatory and anti-inflammatory factors were significantly increased in the brain tissue of post-stroke depressed mice (*p* < 0.01), indicating that depression promoted neuroinflammation. However, exercise decreased proinflammatory factors and further increased the content of anti-inflammatory factors (*p* < 0.05), suggesting that exercise reduces neuroinflammation by modulating inflammatory factor levels.

Nissl staining was employed to observe Nissl bodies and neuronal morphology. As illustrated in Fig. 3e,f, post-stroke depressed mice showed significant reduction in Nissl bodies (*p* < 0.01), with loosely arranged neurons, incomplete cell morphology, and blurred edges. Conversely, the exercise group exhibited a significant increase in hippocampal neurons (*p* < 0.05), with regular cell arrangement, compact structure, and reduced neuronal damage.

Furthermore, neuroinflammation was observed using HE staining (Fig. 3g). The control group had well-organized neuronal cells without any histopathological changes. However, the model group exhibited disorganized neuronal arrangements, numerous vacuolated and apoptotic cells, and inflammatory infiltration, indicative of neuritis. Moreover, in the exercise group, the number of inflammatory cells decreased, and the cellular structure improved significantly. These results indicate that exercise reduces neuroinflammation and alleviates neuronal damage in post-stroke depressed mice.

### Exercise Modulates SIRT1/BDNF1 Signaling Pathway and Improves Synaptic Plasticity

To explore the motor-mediated signaling pathway, SIRT1 knockdown was achieved through adeno-associated virus micro-injection into the mouse hippocampus. AAV vectors containing either SIRT1-shRNA or control shRNA were constructed and injected into the hippocampus of mice in the model+exercise group. The expression levels of SIRT1 and BDNF in the hippocampus of mice were assessed using WB and RT-PCR.

**Fig. 4.**
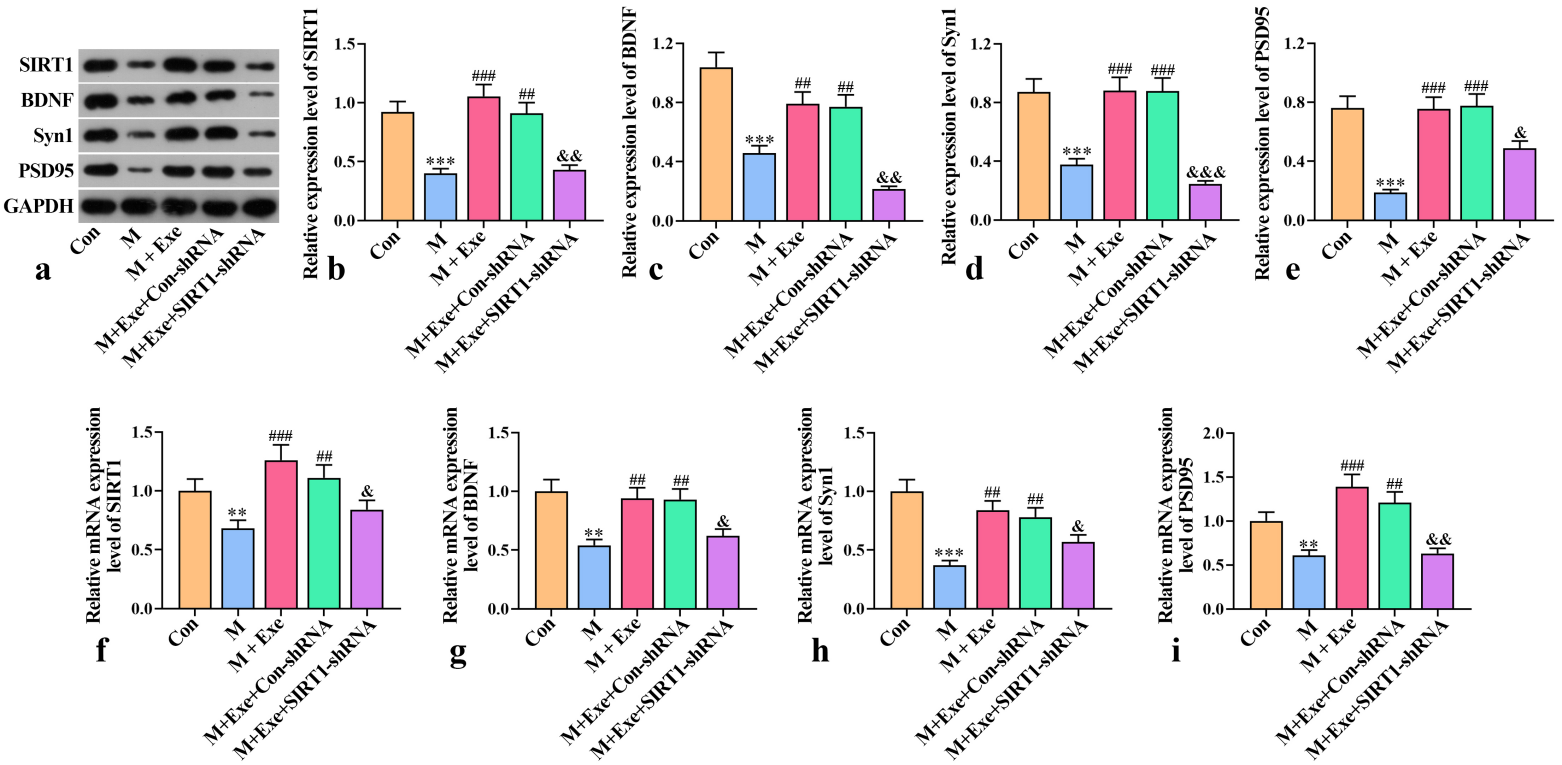
**Effect of exercise on synaptic plasticity (n = 8)**. (a–e) The protein expression levels of SIRT1, BDNF, Syn1, and PSD95 were determined using WB analysis. (f–i) The mRNA expression levels of SIRT1, BDNF, Syn1, and PSD95 were assessed using RT-qPCR. ^*⁣**^p < 0.001, ^**^p < 0.01 versus the control group; ^#⁢#⁢#^*p* < 0.001, ^#⁢#^*p* < 0.01 versus the model group; ^&⁣&&^*p* < 0.001, ^&&^*p* < 0.01, ^&^*p* < 0.05 versus the Con-shRNA group. SIRT1, silent information regulator factor 2-related enzyme 1; BDNF, brain-derived neurotrophic factor; Syn1, synaptophysin; PSD95, postsynaptic density 95; GAPDH, glyceraldehyde-3-phosphate dehydrogenase; WB, western blot; RT-qPCR, reverse transcription-polymerase chain reaction.

As shown in Fig. 4, the expression levels of SIRT1 and BDNF were significantly alleviated in the PSD model group (*p* < 0.01). However, exercise training restored the expression levels of these factors (*p* < 0.01). Moreover, following SIRT1 knockdown, the exercise-induced increase in expression levels of neurotrophic factors was abolished (*p* < 0.05). These results suggest that exercise training reduces depression by increasing neurotrophic factor levels.

Furthermore, analysis of synaptic plasticity markers, Syn1 and PSD95, indicated decreased expression in the PSD model (*p* < 0.01), suggesting impaired synaptic plasticity. Exercise training restored Syn1 and PSD95 expression levels in the model mice, effectively reshaping synaptic plasticity. However, SIRT1 knockout reversed the exercise-induced increase in Syn1 and PSD95 expression (*p* < 0.05). These findings suggest that exercise enhances synaptic plasticity by activating the SIRT1-dependent BDNF signaling pathway.

### Exercise Alleviates Astrocyte Dysfunction in Mice

**Fig. 5.**
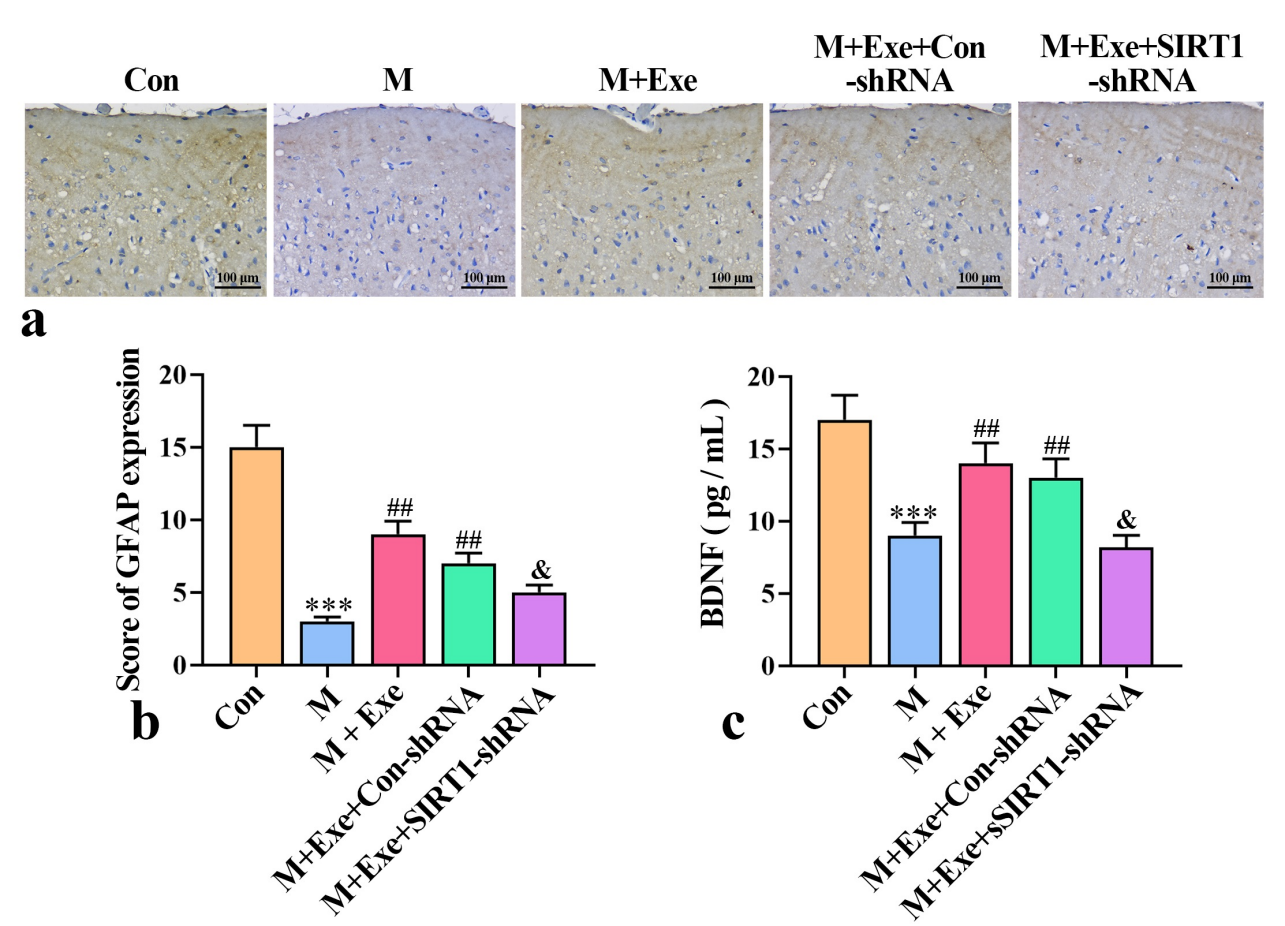
**Effect of exercise on astrocyte function (n = 8)**. (a) Representative images of GFAP-positive cells. (b) Score analysis of GFAP expression intensity in mouse brain. (c) BDNF levels in mouse brain tissue. ^*⁣**^p < 0.001 versus the control group; ^#⁢#^*p* < 0.01 versus model group; ^&^*p* < 0.05 versus the Con-shRNA group. GFAP, glial fibrillary acidic protein.

GFAP, a specific marker of astrocytes, shows their activation state. As shown in Fig. 5a,b, the brown hyper-staining area, indicating the positive rate of GFAP, decreased significantly in the model group (*p* < 0.001), suggesting decreased astrocyte activation. In contrast, the brown hyper-staining rate significantly increased in the M+Exe group (*p* < 0.01), indicating that exercise promotes astrocyte’s functional recovery. However, in the M+Exe group, down-regulation of SIRT1 negated the exercise-induced increase in GFAP expression (*p* < 0.05), underscoring the crucial role of SIRT1 in mediating exercise effect on astrocyte activation.

Under pathological conditions, astrocytes release neuronutrients, maintain synaptic growth, and protect or repair damaged cells. As depicted in Fig. 5c, BDNF activity in the hippocampus of the PSD model group was significantly reduced (*p* < 0.001). However, exercise reversed this decline in BDNF activity (*p* < 0.01). Furthermore, SIRT1 knockdown reduced exercise-induced increase in BDNF activity (*p* < 0.05). These results indicate that SIRT1 is crucial to exercise-induced astrocyte activation and BDNF modulation.

### The BDNF Signaling Pathway Activates mTOR

**Fig. 6.**
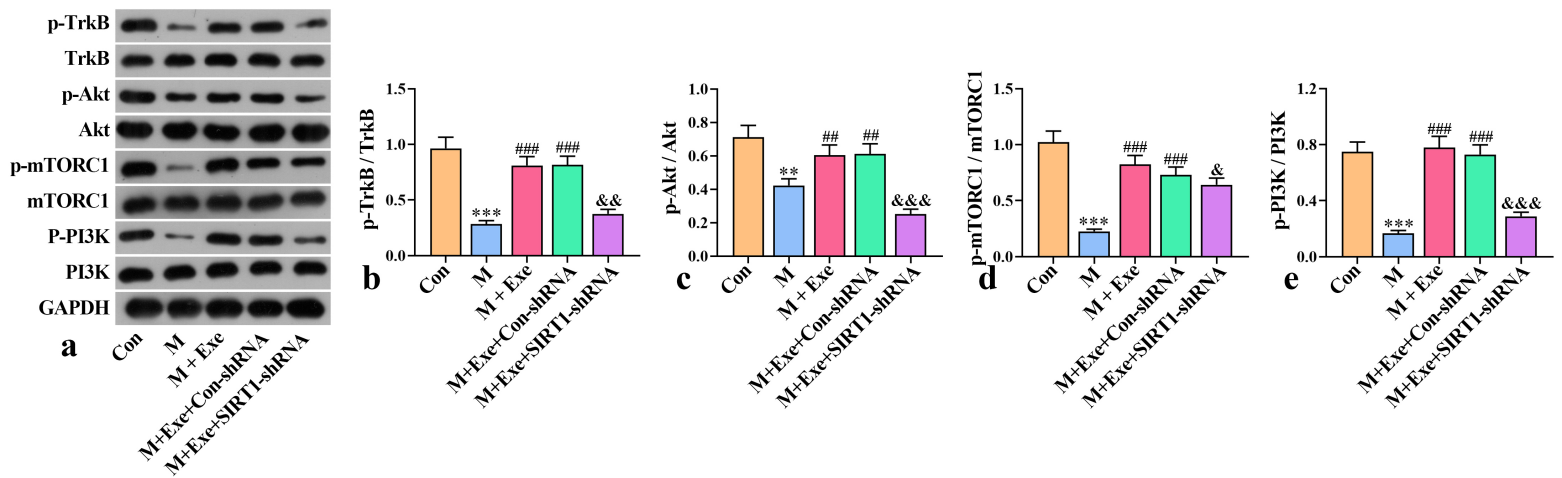
**Effects of exercise on mTOR signaling pathway (n = 8)**. (a) Western blot analysis of mTOR signaling pathway. (b–e) p-TrkB/TrkB, p-Akt/Akt, p-mTORC1/mTORC1, and p-PI3K/PI3K relative protein expression levels. ^*⁣**^p < 0.001, ^**^p < 0.01 versus the control group; ^#⁢#⁢#^*p* < 0.001, ^#⁢#^*p* < 0.01 versus the model group; ^&⁣&&^*p* < 0.001, ^&&^*p* < 0.01, ^&^*p* < 0.05 versus the Con-shRNA group. mTOR, mammalian target of rapamycin; p-TrkB, phosphorylated-tropomyosin receptor kinase B; TrkB, tropomyosin receptor kinase B; p-Akt, phosphorylated-protein kinase B; Akt, protein kinase B; p-mTORC1, phosphorylated-mammalian target of rapamycin complex 1; mTORC1, mammalian target of rapamycin complex 1; p-PI3K, phosphorylated-phosphoinositide 3-kinase; PI3K, phosphoinositide 3-kinase.

The expression levels of p-TrkB/TrkB, p-Akt/Akt, p-mTORC1/mTORC1, and p-PI3K/PI3K in the hippocampus of mice were assessed using WB analysis. As illustrated in Fig. 6, phosphorylation levels of TrkB, Akt, mTORC1, and PI3K were substantially reduced in the model group than in the control group (*p* < 0.01). Exercise intervention increased the phosphorylation levels of these signaling pathways (*p* < 0.01). However, the phosphorylation levels of TrkB, Akt, mTORC1, and PI3K were decreased following SIRT1 knockdown (*p* < 0.05). These results indicate that exercise promotes neuroplasticity by releasing BDNF and activating the TrkB, Akt, and mTOR signaling pathways.

## Discussion

Neuroinflammation is caused by various triggers, such as infection, autoimmune responses, trauma, and hypoxia, and is commonly observed as a secondary effect in neurological conditions like cerebral ischemia and traumatic brain injury [21]. Persistent or uncontrolled inflammatory responses, particularly chronic brain tissue inflammation, can directly or indirectly influence mood and behavior, potentially contributing to neuropsychiatric disorders like depression [22]. Research demonstrates that neuroinflammation leads to neuronal damage, disrupts neuronal survival and function, and impacts the activity of immune cells within the nervous system, specifically microglia and astrocytes [23]. This cascade results in neurotoxicity, further exacerbating both neuroinflammation and depression-like behaviors. Consequently, targeting neuroinflammation to alleviate depression-like behaviors has emerged as a viable therapeutic approach. Exercise, a safe, effective, and cost-efficient non-pharmacological intervention, has shown promise in managing depression. However, the precise mechanism of exercise reducing neuroinflammation in chronically stressed mice models remains unclear.

In this study, we established a MCAO+CUMS mouse model and subjected these model mice to a regular exercise training regimen. Initially, we evaluated neurological deficits and observed that MCAO+CUMS treatment induced substantial neural abnormalities, which were alleviated by regular exercise. Then, we assessed the inflammatory state of the brain tissue in the MCAO+CUMS mice, identifying pathological changes, including decreased hippocampal neuron count, disrupted nerve function, and increased levels of inflammatory factors, such as IL-1β and TNF-α. Neuroinflammation triggers the activation of microglia, the immune cells of the brain, which contributes to the development of depression by producing pro-inflammatory cytokines such as IL-1β, TNF-α, and IL-6 [24]. These cytokines negatively impact synaptic plasticity, leading to depression-like behaviors and mood disorders [25]. Exercise training significantly reduced the levels of inflammatory factors in the hippocampus, and restored the morphological structure of the neurons. These results indicate that exercise mitigates depression-like behaviors in mice and alleviates neuroinflammation and neuronal damage in the brain.

To investigate the impact of exercise on neuronal function, we examined neuroplasticity. Neuroplasticity refers to the nervous system’s ability to modify its structure, function, and connections in response to internal or external stimuli [26]. Under normal conditions, astrocytes secrete neurotrophic factors, such as BDNF, nerve growth factor (NGF), glial cell line-derived neurotrophic factor (GDNF), and neurotrophin (NT) 4/5, which provide essential support for neurons and maintain neuroplasticity [27]. In neuroinflammatory states, astrocyte depletion decreases the secretion of neurotrophic factors, disrupting mechanisms like synaptic plasticity and neurogenesis, contributing to the manifestation of depression-like behaviors [28].

Previous studies reveal that SIRT1 regulates BDNF expression. SIRT1 deficiency results in upregulation of microRNA-134, which directly inhibits the translation of cyclic AMP-response element binding protein (CREB) mRNA, thereby down-regulating BDNF expression [29, 30]. In contrast, SIRT1 over-expression in the hippocampus cornu ammonis1 (CA1) region increases the expression of SIRT1 and BDNF, restores synaptic plasticity, enhances neuronal excitability, and improves cognitive impairment [31]. Exercise, which alters energy demands and levels of high-energy molecules such as adenosine triphosphate (ATP) and coenzyme nicotinamide adenine dinucleotide hydrate (NADH), enhances NAD^+^ synthesis or increases the NAD^+^/NADH ratio, thereby boosting SIRT1 activity [32].

Therefore, we used an adeno-associated virus to down-regulate SIRT1 in mice from the M+Exe groups. The results showed that the levels of SIRT1, BDNF and synaptic marker proteins such as PSD95 and Syn1 significantly decreased in the PSD model group, suggesting impaired synaptic plasticity. However, exercise increased the expression of SIRT1, BDNF, PSD95, and Syn1, restoring neuroplasticity. Moreover, SIRT1 knockdown in the M+Exe group reduced these exercise-induced increases. This suggests that exercise intervention promotes neuronal growth and synaptic plasticity by activating the SIRT1-mediated BDNF signaling pathway, enhancing the brain’s self-repair capabilities. We further validated these findings using GFAP positive tests, which revealed that the PSD model reduced astrocyte numbers and triggered structural atrophy, thereby disrupting astrocyte function. However, exercise increased astrocyte counts and restored their functionality, further validating the role of exercise in improving neuroplasticity.

The mTOR pathway is closely associated with the manifestation of depression-like behaviors, including anxiety, depressive symptoms, and neuronal atrophy [33]. TrkB, a functional receptor for BDNF, is activated by BDNF, inducing the PI3K/Akt/mTORC1 cascade. This signaling pathway promotes dendritic growth and BDNF secretion, which is crucial role in regulating synaptic transmission and long-term potentiation, thereby improving synaptic plasticity [34]. Chronic stress has been reported to decrease hippocampal Akt and extracellular signal-regulated kinase (ERK) phosphorylation, impairing hippocampal neuroplasticity by downregulating mTORC1 signaling [35]. However, exercise intervention increases PI3K/Akt phosphorylation, increases mTORC1 phosphorylation and activates mTOR. mTOR activation promotes neural maturation, synaptic formation, and synaptic plasticity, contributing to its antidepressant effects. Despite promising findings, this study has certain limitations. Only mouse tissues were examined, and no cellular experiments were performed, which may introduce biases due to individual differences or the effect of adenovirus action on multiple cells. Hence, cellular level investigations are needed to validate these results. Secondly, we employed a combination of stroke and CUMS to establish a PSD model. However, stroke primarily influences emotional states through mechanisms such as neural damage, inflammatory responses, and neurotransmitter imbalance, whereas CUMS induces depressive-like behaviors via prolonged psychosocial stress. This experimental design may therefore make it difficult to clearly distinguish the independent contributions of stroke and CUMS to depressive-like behaviors, thereby limiting our in-depth understanding of the pathological mechanisms underlying PSD. Furthermore, while this study primarily focuses on the hippocampus, PSD also causes pathological changes in the brain tissues such as the amygdala and prefrontal cortex [36]. Future studies should observe cellular changes in these additional brain regions.

## Conclusion

Regular exercise reduces post-stroke depressive-like behaviors in mice by decreasing brain inflammatory factor levels, reducing neuroinflammation, and potentially improving synaptic plasticity and astrocyte activation through the SIRT1-BDNF-mTORC1 signaling pathway.

## Availability of Data and Materials

The data analyzed was available upon the request from the corresponding author.
